# DDIAS, DNA damage-induced apoptosis suppressor, is a potential therapeutic target in cancer

**DOI:** 10.1038/s12276-023-00974-6

**Published:** 2023-05-01

**Authors:** Joo-Young Im, Mi-Jung Kang, Bo-Kyung Kim, Misun Won

**Affiliations:** 1 Personalized Genomic Medicine Research Center, KRIBB, Daejeon, 34141 Republic of Korea; 2 University of Science and Technology (UST), Daejeon, 34113 Republic of Korea; 3 R&D Center, OneCureGEN Co., Ltd., Daejeon, 34141 Republic of Korea

**Keywords:** Oncogenes, Drug development

## Abstract

Increasing evidence indicates that DNA damage-induced apoptosis suppressor (DDIAS) is an oncogenic protein that is highly expressed in a variety of cancers, including colorectal cancer, lung cancer, breast cancer, and hepatocellular carcinoma (HCC). The discovery of DDIAS as a novel therapeutic target and its role in human cancer biology is fascinating and noteworthy. Recent studies have shown that DDIAS is involved in tumorigenesis, metastasis, DNA repair and synthesis, and drug resistance and that it plays multiple roles with distinct binding partners in several human cancers. This review focuses on the function of DDIAS and its regulatory proteins in human cancer as potential targets for cancer therapy, as well as the development and future prospects of DDIAS inhibitors.

## Cancer: tumour-protecting protein targeted for new therapies

Inhibiting the activity of a protein that protects cancer cells against apoptosis, normal programmed cell death, could provide therapeutic approaches for multiple cancers. The DNA damage-induced apoptosis suppressor (DDIAS) protein is over-expressed in cancers, and plays key roles in tumour development, metastasis and drug resistance. Misun Won, Joo-Young Im and co-workers at the Personalized Genomic Research Center in Daejon, South Korea, reviewed current understanding of the function of DDIAS. DDIAS promotes cellular proliferation and plays a significant role in advanced tumour metastasis in lung cancer, breast and liver cancer cells. The protein protects lung cancer cells from being destroyed by DNA damage agents. Knocking out DDIAS in lung cancer cells enables apoptosis, and DDIAS inhibitors are showing promise in early trials. Further work is needed to fully understand DDIAS functioning.

## Introduction

DNA damage-induced apoptosis suppressor (DDIAS) was first discovered as a human homolog (*hNoxin*) of mouse *noxin* via genomic analysis of colorectal cancer patients and large-scale siRNA screening aimed at searching for cancer-related genes^1,2^. DDIAS was named based on its antiapoptotic properties in response to DNA repair in cancer cells.

DDIAS is highly expressed in several human cancers, including colorectal cancer, lung cancer, breast cancer and hepatocellular carcinoma (HCC), and stimulates cancer cell proliferation and cell cycle progression^2–5^. DDIAS plays a vital role in tumorigenesis, metastasis, DNA repair and drug resistance by inhibiting cell death mediated by DNA damage agents, tumor necrosis factor-related apoptosis-inducing ligand (TRAIL) and gefitinib in lung cancer^2,5–8^. In HCC, DNA copy number amplification of DDIAS has been observed^3^. Interestingly, DDIAS interacts with various binding partners to drive numerous processes in the membrane, cytoplasm and nucleus through various extracellular signals.

Although DDIAS studies on the novel cancer-related processes are still lacking, DDIAS appears to play a key role in carcinogenesis, particularly in lung, liver and colorectal cancers. DDIAS is not a well-known gene to most cancer researchers even though it is noteworthy as a novel cancer therapeutic target. In this review, we discuss various aspects of DDIAS, including transcriptional regulation, degradation, DNA repair, and resistance to apoptosis, and suggest prospective cancer treatments by inhibiting DDIAS-related cellular functions.

## Molecular features of DDIAS

**Fig. 1:**
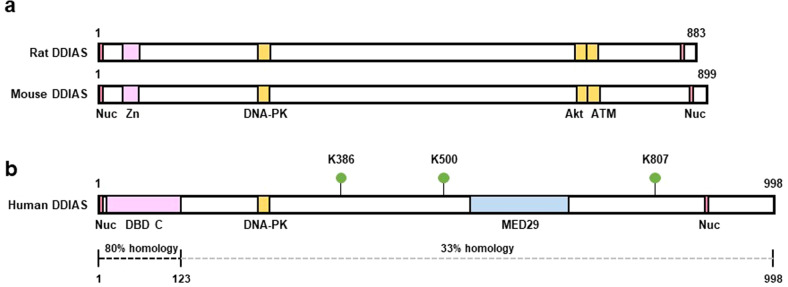
Schematic representation of the DDIAS protein. **a**, **b** Comparison of rat, mouse, and human DDIAS proteins. Sequence comparison of human DDIAS protein with mouse DDIAS (noxin) revealed high homology in the N-terminal region containing the DBD C (80.5%; amino acids 1–123) and less homology throughout the rest of the proteins (33.2%; amino acids 124–998). Nuc, nuclear localization signals (red); Zn, zinc-finger-like domain (pink); DNA-PK, DNA-PK phosphorylation consensus sequences (yellow); Akt or ATM, Akt or ATM phosphorylation consensus sequences (yellow); DBD C, DNA binding domain C (Pink); MED29, mediator complex subunit 29 (blue). Potential ubiquitination sites in DDIAS (K386, K500, and K807) are shown.

The *DDIAS* gene (noxin, C11orf82, GeneID220042) encodes 998 amino acids and consists of six exons with a translation initiation site at the third exon. DDIAS features a DNA-binding domain C (DBD C) in the N-terminal region (amino acids 8–123), which is also found in replication protein A (RPA), a nuclear single-stranded DNA-binding protein. DBD C is essential for forming heterotrimeric complexes with RPA1 with RPA2 and RPA3 for replication, recombination and repair, and interactions with nuclear proteins^9–11^. The *DDIAS* gene is located on human chromosome 11 and is conserved (70% similarity at the DNA level) in the rat and mouse genomes (rat chromosome 1 and mouse chromosome 7). Although DDIAS has been recognized as a human homolog of mouse nitric oxide-inducible (noxin), a sequence comparison of the DDIAS protein with mouse noxin reveals high homology only in the N-terminal region containing the DBD C (80.5%; amino acids 1–123) and lower homology throughout the remainder of the protein sequences (33.2%; amino acids 124–998), despite conserved several sequences (Fig. 1). The DDIAS protein sequence is enriched with serine (132 serine residues), indicating that phosphorylation of these residues might be a potential modification. Similar to mouse noxin, DDIAS carries putative sites of phosphorylation by DNA-PK, ATM, cdc2, CDK5, CKII, p38 mitogen activated protein kinase (p38MAPK), RSK, PKA, and PKC (NetPhos 3.1). Recently, Akimov and colleagues reported that DDIAS is ubiquitinated at lysine386, lysine500 and lysine807, as previously discovered using the UbiSite method^12^ (Fig. 1).

## Regulation of DDIAS expression

### DDIAS expression patterns

In normal human tissues, low expression of DDIAS has been detected in the lung, stomach, thymus, colon and heart, while high expression has been detected in the human testis, pancreas and prostate^2^. However, DDIAS mRNA levels are significantly higher in lung, breast, and colorectal cancer tissues and cancer cell lines than in normal tissues or cells^2,3^. HCC is characterized by DDIAS overexpression with DNA copy number amplification on chromosome 11q14.1^5^. DDIAS expression is induced by ultraviolet (UV) irradiation, and its level is highest in the S phase of the cell cycle in cancer and normal cells. Similar to DDIAS, mouse noxin is highly expressed in the testis^1,2^. Similarly, numerous stressors, such as gamma ray irradiation, UV irradiation, NO donors, hydrogen peroxide, adriamycin, and cytokines, stimulate the production of mouse noxin. However, mouse noxin expression is highest in cells in the G2/M phase or after exposure to nocodazole, a G2/M arrest inducer.

The Human Protein Atlas revealed that DDIAS is found mainly in the cytoplasm. Endogenous DDIAS is present in the cytoplasm of lung cancer cells and tissues, as well as the nucleus of HCC cells^4,5,13^. In NIH3T3 cells, endogenous mouse noxin is found in the cytoplasm and the nucleus; however, it accumulates in the nucleus in response to exposure to the nitric oxide donor SNAP^1^.

### Transcriptional regulation of DDIAS

**Fig. 2:**
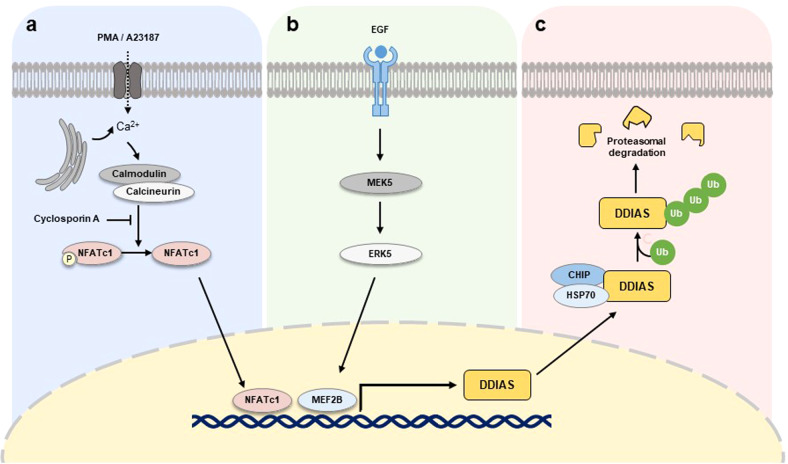
Regulation of DDIAS expression. **a** Activated NFAT2 regulates DDIAS transcription. **b** In response to EGF, MEK5/ERK5/MEF2B pathways induce DDIAS expression. **c** DDIAS ubiquitination mediated by HSP70/CHIP regulates DDIAS protein turnover.

*DDIAS* is a target gene of nuclear factor of activated T cells 1 (NFATc1, NFAT2) in lung cancer^6^. In a *DDIAS* promoter analysis, potential binding sites for various transcription factors, such as p300, SP1, C/EBP, or NFAT, were identified. Among these transcription factors, dephosphorylated NFATc1 activates the transcription of *DDIAS* by binding to NFAT consensus sequences in the DDIAS promoter. DDIAS gene expression is stimulated by phorbol 12-myristate 13-acetate and the calcium ionophore A23187 and suppressed by the calcineurin inhibitor cyclosporin A, which activate and inhibit NFATc1, respectively (Fig. 2a). Additionally, tissue array immunostaining revealed a correlation between DDIAS and NFATc1 expression in human lung cancers^6^. Despite the importance of the NFAT family in the immune response, recent studies have indicated that activation or overexpression of NFATc1 in human solid tumors and hematological malignancies is associated with tumor progression^14,15^.

Although DDIAS expression is induced by UV irradiation of normal and cancer cells^2^, the mechanism underlying DDIAS-mediated transcriptional regulation by UV irradiation is not fully understood. UV irradiation generally stimulates mitogen-activated protein kinase (MAPK) and ATM pathways and the activation of transcription factors such as p53, NF-kB, AP-1, NFAT, and Nrf2^16,17^. Similarly, the induction of mouse noxin function mediated by stress stimuli, including UV irradiation, depends on p53^1^.

In addition to UV exposure, DDIAS expression is induced by serum or epidermal growth factor (EGF)^2,13^. Previous studies have demonstrated that extracellular signal-regulated kinase 5 (ERK5) phosphorylates myocyte enhancer factor-2 (MEF2) family proteins and serum glucocorticoid-inducible kinase, all of which are essential for entry into the S phase of the cell cycle^18,19^. DDIAS expression is induced by ERK5 and MEF2 in response to EGF^13,20^. Genetic or pharmacological inhibition of ERK5 suppresses DDIAS expression by EGF exposure. The overexpression of constitutively active MEK5 enhances DDIAS expression (Fig. 2b). In chromatin immunoprecipitation (ChIP) assays, MEF2B (a downstream target of ERK5) exhibited sequence-specific binding to the MEF2-binding site in the DDIAS promoter after EGF treatment. Moreover, overexpression of MEF2B increased the EGF-mediated induction of DDIAS expression, whereas knockdown of MEF2B attenuated this effect.

### Posttranslational regulation of DDIAS

DDIAS stability is regulated by E3 U-box-dependent ubiquitin ligase carboxyl terminus of HSP70-interacting protein (CHIP)-mediated proteasomal degradation^21^. We first identified CHIP as an interacting partner of DDIAS by yeast two-hybrid screening. E3 ubiquitin ligase CHIP physically associates with both the N- and C-terminal regions of DDIAS, allowing this protein to be targeted for proteasomal degradation and thereby reducing the DDIAS half-life in the cytoplasm (Fig. 2c). CHIP deletion study demonstrated a tetratricopeptide repeat (TPR) domain and the U-box are essential for DDIAS ubiquitination. HSP70-bound DDIAS is recruited to the CHIP E3 ligase via the TPR domain, suggesting that DDIAS is a client protein of HSP70. Since CHIP is a chaperone-associated U box-containing E3 ligase, it depends on Hsp70/Hsp90 chaperones. These chaperones interact with oncogenic clients such as c-Myc, hypoxia inducible factor 1a (HIF-1a), NF-kB/p65, and DDIAS^21–24^, implying that CHIP is a tumor suppressor.

## Molecular mechanisms of DDIAS in Cancers

**Fig. 3:**
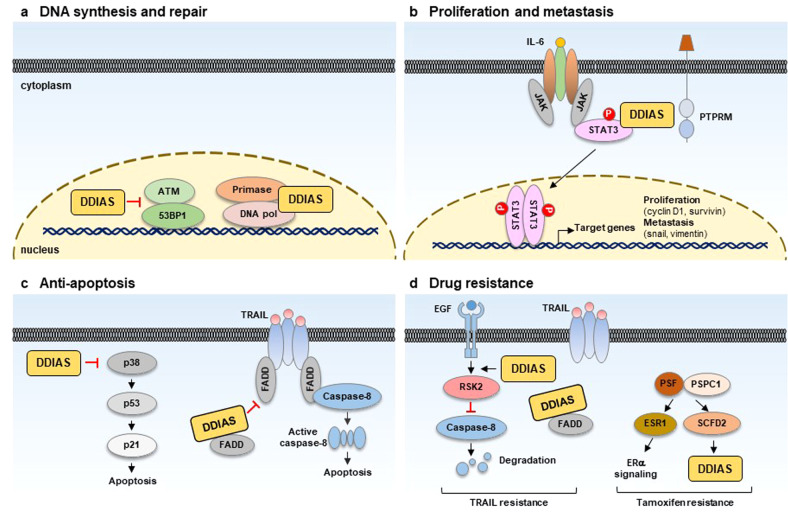
Oncogenic functions of DDIAS mediated via multiple pathways. **a** DDIAS promotes DNA synthesis and DSB (double-strand break) repair in human cancers. **b** DDIAS enhances the proliferation and metastasis of cancer cells mediated by STAT3 phosphorylation by competing with PTPRM. **c** DDIAS prevents cancer cells from undergoing apoptosis by inhibiting the p38 MAPK/p53/p21 pathway and TRAIL-mediated DISC formation. **d** DDIAS contributes to TRAIL and tamoxifen resistance.

DDIAS executes a variety of cellular tasks with its different binding partners (Table 1). High expression of DDIAS in cancer contributes to malignancies mediated via a variety of mechanisms (Fig. 3). The functions of DDIAS associated with cancer are discussed herein.

**Table 1:** Proteins interacting with DDIAS.

Gene symbols	Protein ID	Protein function	Cancer type	Reference
POLA1	DNA polymerase α	DNA synthesis	HCC	Zhang et al.^5^
PRIM2A	Primase	DNA synthesis	HCC	Zhang et al.^5^
STUB1	CHIP	Ubiquitination	NSCLC	Won et al.^21^
HSPA4	HSP70	Chaperone	NSCLC	Won et al.^21^
FADD	FADD	Apoptosis	HCC, NSCLC	Im et al.^7^
STAT3	STAT3	Transcription of target genes	NSCLC	Im et al.^8^
PTPRM	PTPRM	Dephosphorylation	NSCLC	Im et al.^8^

### DNA synthesis and repair

DDIAS overexpression accelerates the G1-S phase transition by enhancing DNA synthesis in HCC^5^. DDIAS interacts with DNA polymerase α, suggesting that DDIAS may boost de novo DNA synthesis by promoting the formation of DNA polymerase-primase complexes (Fig. 3a). DDIAS overexpression promotes cellular proliferation, colony formation, cellular migration and in vivo tumorigenicity, whereas DDIAS knockdown attenuates these effects.

On the basis of computational approaches such as evolutionary rate covariation, a recent study revealed 17,487 mammalian genes coevolved in six distinct DNA repair pathways. Among these coevolved proteins, DDIAS was identified as a novel factor in double-strand break (DSB) repair based on its coevolution with homologous recombination (HR)^25^. It is involved in a DSB repair mechanism mediating nonhomologous end-joining^26,27^. DDIAS depletion resulted in DSB accumulation, as indicated by ATM kinase activation and 53BP1 foci induction, and defective HR (Fig. 3a). Similarly, a previous report showed an increase in H2AXγ, a marker of DNA DSBs, and comet formation, a measure of DNA strand breaks, with cells depleted of DDIAS^2^. Furthermore, DDIAS carries an oligonucleotide/oligosaccharide-binding-fold domain similar to the single-strand DNA-binding domain of RPA^2^. This domain is required for replication, recombination and repair processes such as HR^28^, providing evidence supporting the involvement of DDIAS in DNA repair.

### Proliferation and metastasis of cancer cells

The activation of signal transducer and activator of transcription 3 (STAT3) plays a critical role in cancer cell proliferation, survival, metastasis, and self-renewal^29–32^. Phosphorylated STAT3 enters the nucleus and induces the transcription of target genes, including survivin, Bcl-2, Mcl-1, c-Myc, cyclin D1, slug, and matrix metalloproteinase-2^33–36^. A recent report discovered the protein tyrosine phosphatase receptor mu (PTPRM), a novel PTPase of STAT3^8^. DDIAS promotes tyrosine phosphorylation of STAT3, which is constitutively activated in malignant cancers. DDIAS binds to the STAT3 transactivation domain, competing with PTPRM to recruit STAT3 for dephosphorylation. Indeed, DDIAS inhibits PTPRM/STAT3 binding and STAT3 Y705 dephosphorylation, allowing STAT3 activation to persist in lung cancer (Fig. 3b). Interestingly, DDIAS expression is highly correlated with STAT3 phosphorylation in human lung cancer cell lines and tissues, regardless of PTPRM expression^8^, and is considered a potential biomarker and therapeutic target in malignant lung cancer cells with aberrant STAT3 activation. Given that STAT3 plays a crucial role in the immune response^37,38^ and that DDIAS is highly expressed in bone marrow and spleen^1^, the next challenge will be the investigation of DDIAS function in the immune response against infection and the regulation of its expression in immune cells.

DDIAS expression is significantly linked with advanced tumor-node-metastasis stage in breast cancer and positive regional lymph node metastasis in NSCLC patients^3,4^. DDIAS positively regulates the protein levels of β-catenin and snail in HeLa and NSCLC cells treated with EGF, promoting cancer cell invasion^13,20^. Apparently, DDIAS activates STAT3 and promotes migration and invasion by expressing genes such as survivin, slug, and vimentin in response to interleukin-6 (IL-6) in lung cancer cells^8^.

### Antiapoptotic function

A previous study demonstrated that DDIAS exhibits an antiapoptotic function in lung cancer in response to DNA damage^2^. DDIAS protects lung cancer cells against apoptosis in response to DNA damage agents such as camptothecin, cisplatin, and UV irradiation in lung cancer cells^2,6,21^. Depleting DDIAS inhibited the proliferation of lung, breast, and hepatoma cancer cells in vitro and in vivo^2,3,5^. Moreover, studies have shown that DDIAS knockdown triggered apoptosis in A549 non-small cell lung cancer (NSCLC) cells by activating p38MAPK/p53/p21^2^ (Fig. 3c). Additionally, NFATc1 knockdown or CHIP overexpression, which resulted in a decrease in DDIAS levels, promoted apoptosis and inhibited tumor development in lung cancer^6,21^ (Fig. 2). In contrast, NFATc1 or DDIAS overexpression protected NSCLC cells against DNA damage agent-mediated cell death and caspase-3/7 activation. Therefore, targeting DDIAS or NFATc1 inhibits the mechanism(s) involved in regulating cisplatin resistance in lung cancer cells.

Remarkably, DDIAS depletion makes NSCLC and HCC cells more susceptible to TRAIL-mediated apoptosis in two different ways^7^ (Fig. 3c). First, the N-terminus of DDIAS binds to the death effector domain of the Fas-associated protein death domain (FADD) and inhibits its recruitment to the death-inducing signaling complex (DISC), thereby inhibiting caspase-8 activation. Second, DDIAS knockdown suppresses EGF-induced phosphorylation of p90 ribosomal S6 kinase 2 (RSK2) and stabilizes caspase-8 by preventing its ubiquitination and proteasomal degradation (Fig. 3d). Therefore, DDIAS exhibits dual functions in inhibiting DISC formation, thereby suppressing TRAIL-mediated apoptosis of cancer cells.

### Induction of drug resistance

The paraspeckle component 1 (PSPC1)-Sec1 family domain containing 2 (SCFD2)-DDIAS axis is highly expressed in tamoxifen-resistant breast cancer and is a potential diagnostic and therapeutic target for estrogen receptor (ER)-positive breast cancer^39,40^. PSPC1 is an RNA-binding protein (RBP) belonging to the Drosophila behavior human splicing (DBHS) family and functions as a cancer transcriptional regulator^41,42^. PSPC1 interacts with a splicing factor known as polypyrimidine tract-binding protein-associated splicing factor (PSF). PSF is another RBP in the DBHS family that controls the action of target genes such as SCFD2 and estrogen receptor 1^40^. Furthermore, the antiapoptotic genes DDIAS and MYBL1 are downstream target genes of SCFD2 in ER-positive breast cancer cells (Fig. 3d). DDIAS may be a potential therapeutic target for tamoxifen-resistant breast cancer since the inhibition of PSPC1 or SCFD2 reduces in vivo tumor development in tamoxifen-resistant breast cancer.

Several types of cancer cells, including breast cancer, NSCLC and HCC cells, exhibit TRAIL resistance, leading to the dysfunction of the death receptors DR4 and DR5, a defect in DISC assembly, and the high expression of apoptosis inhibitors^43,44^. Therefore, DDIAS contributes to TRAIL resistance by inhibiting DISC formation through FADD binding and inducing caspase-8 degradation in NSCLC and HCC cells^7^ (Fig. 3d).

## Development of DDIAS inhibitors for cancer therapy

Our group and other teams have explored the regulatory mechanism of DDIAS expression and various functions involved in carcinogenesis. DDIAS overexpression promotes the progression of lung cancer, colon cancer, breast cancer, and HCC through various mechanisms, including DNA synthesis and repair, p53 signaling proliferation and metastasis, STAT3 activation, death ligand signaling, and drug resistance (Fig. 3). Therefore, it has been proposed that anticancer treatments can be developed by suppressing the oncogenic function of DDIAS. Genetic or pharmacologic inhibition of DDIAS can be explored for anticancer drug development.

### DDIAS nucleotide-based therapies using small interfering RNAs (siRNAs) or antisense oligonucleotides (ASOs)

Oligonucleotides such as miRNAs, siRNAs, ASOs and synthetic mRNAs show the potential to modulate gene expression via RNAi, RNase H-mediated cleavage, splicing modulation, noncoding RNA inhibition, gene activation and programmed gene editing^45,46^. Recently, significant progress has been made in oligonucleotide delivery through chemical modifications such as the P=S bond, 2’-OMe, 2-OME, LNA, PNA, and PMO, and cell targeting technology based on GalNac, GLP receptor, folate receptor and transferrin receptor, and nanoparticle carriers^45–48^. These developments provide cutting-edge technology for creating DDIAS siRNAs or ASOs for cancer treatment.

Recently, four siRNA therapeutics, ONPATTRO (patisiran), GIVLAARI (givosiran), OXLUMO (lumasiran) and LEQVIO (Inclisiran), and eleven ASO therapeutics, including Spinraza (nusinersen), Tegsedi (inotersen), and Waylivra (volanesorsen), received FDA approval, and many drugs have been entered into clinical trials^45,46^. Although oligonucleotide-based anticancer drugs have not been FDA approved, the development of many anticancer ASO and siRNA drugs is underway.

**Fig. 4:**
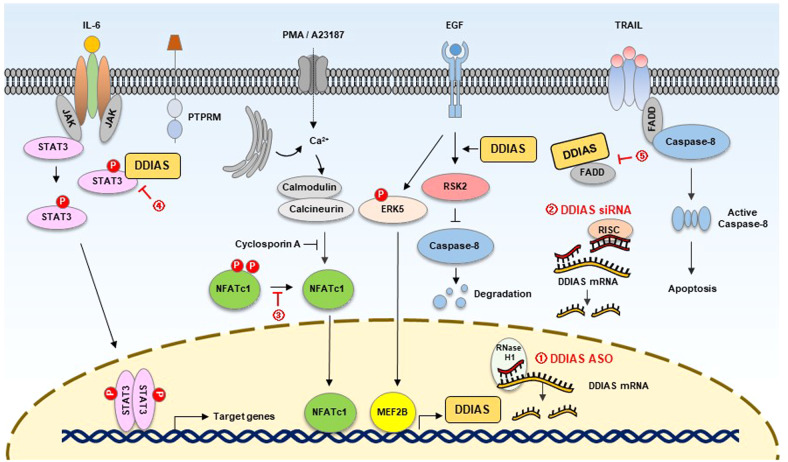
Proposed strategies for inhibiting DDIAS in cancer. (1) Inhibition of DDIAS levels by ASOs; (2) inhibition of DDIAS levels by siRNAs; (3) inhibition of DDIAS transcription by regulating NFATc1 dephosphorylation; (4) inhibition of DDIAS/STAT3 binding; and (5) suppression of DISC formation by the inhibition of DDIAS/FADD binding.

Because structural modification of DDIAS involved in the mechanism of carcinogenesis and malignancy is not clearly understood, the first approach to develop anticancer agents based on DDIAS is to generate optimal siRNAs or ASOs (Fig. 4). Previously, siRNA-mediated DDIAS knockdown resulted in efficient anticancer activity in vitro and in vivo^2,5^.

### Inhibitors of DDIAS transcription

The mechanisms underlying the transcriptional regulation and degradation of DDIAS have been identified (Fig. 2). A recent study discovered a quinoxaline derivative, DGG-100629, that blocks DDIAS transcription^49^. DGG-100629 induces c-Jun NH(2)-terminal kinase (JNK) activation and inhibits NFATc1 nuclear translocation (Fig. 4). JNK1 inhibition genetically or pharmaceutically restores the suppression of DDIAS expression and cell death caused by DGG-100629. As expected, DGG-100629-induced lung cancer cell death was reversed by overexpressing DDIAS or STAT3. In a xenograft assay, DGG-100629 inhibited tumor growth by lowering STAT3 activity and the expression of its target genes. Moreover, DGG-100629 reduced the proliferation of gefitinib-resistant lung cancer cells isolated from patients who expressed high levels of NFATc1 and DDIAS, suggesting that blocking DDIAS transcription is a unique approach for the treatment of gefitinib-resistant lung cancer. Additionally, inhibitors of NFATc1, ERK5, MEF2B, calmodulin, and calcineurin that are involved in DDIAS transcription can be developed as anticancer agents.

### Inhibitors of the STAT3-DDIAS interaction

The association of STAT3 with tumorigenesis and immune functions has been extensively investigated in numerous cancers for decades^30,37^. Targeting the STAT3 signaling pathway has been used as a promising therapeutic strategy in cancer, and preclinical and clinical studies on STAT3 inhibitors are ongoing^32,50–52^.

DDIAS competes with PTPRM to bind to STAT3, allowing STAT3 Y705 phosphorylation to persist in lung cancer. The inhibition of DDIAS/STAT3 binding can exert an anticancer impact by decreasing STAT3 activity (Fig. 4). After screening a chemical library of 11,211 compounds, researchers identified miconazole, an antifungal agent, as an inhibitor of the DDIAS/STAT3 interaction^53^. Notably, the interaction between DDIAS and STAT3 disappeared in the presence of miconazole, which suppressed STAT3 tyrosine Y705 phosphorylation and the expression of its target genes. Miconazole inhibited the growth, migration and invasion of lung cancer cells^53^. In an NCI-H1703 mouse model, miconazole significantly suppressed tumor size, decreasing the phosphorylation of STAT3 Y705 and the expression of its targets, such as cyclin D1, survivin, and snail. Novel inhibitors of the DDIAS/STAT3 interaction can be developed as anticancer agents.

## Role of DDIAS in other diseases

For the most part, DDIAS has been considered an oncogene in various cancers. DDIAS is one of the upregulated genes epigenetically modified in the prefrontal cortex of adult mice with a history of early life stress and is implicated in long-lasting behavioral abnormalities caused by early life stress^54^. Additionally, DDIAS was one of the cell cycle regulatory genes altered in skin fibroblasts in classical Ehlers‒Danlos syndrome patients harboring a pathogenic *COL5A1* and *COL5A2* gene variant encoding type V collagen (COLL V)^55^. A recent study found that environmental pollutants, including 1,4-dioxane, boosted DDIAS expression to trigger the DNA damage and repair response in mouse liver^56^. These findings based on genomic and transcriptomic analyses provide the opportunity to unveil various functions of DDIAS in brain development and other diseases in addition to cancers.

## Perspectives

Despite its large size and low secondary structure expansion, DDIAS has been identified as a potential therapeutic target for a variety of cancers. DDIAS is clearly implicated in various oncogenic pathways and causes human cancer by linking multiple networks through its interactions with many nuclear and cytoplasmic binding partners. However, more studies are still required to establish DDIAS as a therapeutic target. Specifically, 1) elucidation of signaling network involved in the regulation of expression, activation and the distribution of DDIAS in the nucleus and cytoplasm; 2) domain study involving posttranslational modifications, such as phosphorylation, methylation, and acetylation and exploring DDIAS function and subcellular localization and; and 3) further investigation of DDIAS-binding partners involved in carcinogenesis are needed.

Based on the recent DDIAS studies, the strategy for developing anticancer drugs involves the development of the following agents: 1) DDIAS-reducing agents such as siRNAs, shRNAs, and ASOs; 2) direct or indirect transcriptional inhibitors of DDIAS, such as proteins that dephosphorylate NFAT2 or ERK5; 3) inhibitors of cancer-related DDIAS functions, such as DDIAS-STAT3 binding and DDIAS-FADD binding; 4) RSK2 inhibitors that cleave caspase 8; and 5) anticancer agents developed on the basis of cancer-related DDIAS function.

Interestingly, high expression of DDIAS correlates with that of STAT3 in bone marrow and spleen, suggesting that DDIAS is involved in the immune response. Additionally, upregulation of DDIAS in the prefrontal cortex of adult rats subjected to stress at a young age explains a role for DDIAS in the stressed brain. More DDIAS studies and others may reveal pathological characteristics and mechanisms associated with cancer as well as other diseases.
